# Percutaneous endoscopic lumbar discectomy for single and double segment lumbar disc herniation with sciatic scoliosis in adults: a retrospective study

**DOI:** 10.1186/s12893-024-02314-5

**Published:** 2024-01-31

**Authors:** Jitao Yang, Haopeng Luan, Jiawei Ren, Jiyuan Tao, Weibin Sheng, Hailong Guo, Qiang Deng

**Affiliations:** Department of Spine Surgery, The First Affiliated Hospital of Xinjiang Medical University, Urumqi, Xinjiang 830054 China

**Keywords:** Sciatic scoliosis, Adult lumbar disc herniation, Single segmental, Double segmental, Percutaneous endoscopic lumbar discectomy

## Abstract

**Objective:**

Sciatic scoliosis can be seen in patients with lumbar disc herniation. Percutaneous endoscopic lumbar discectomy (PELD) is a common surgical method for the treatment of lumbar disc herniation. The difference between single-segment lumbar disc herniation and double-segment lumbar disc herniation with Sciatic Scoliosis in adults after PELD needs further study. The aim of this study was to compare the imaging features of single-segment and double-segment lumbar disc herniation with Sciatic Scoliosis in adults and to further explore the clinical outcomes of functional improvement and scoliosis imaging parameters of the two groups after PELD.

**Methods:**

Adult patients with lumbar disc herniation with sciatic scoliosis who received PELD from January 2019 to June 2022 were analyzed retrospectively. According to the number of operative segments, the patients were divided into a single-segment group and a double-segment group. Perioperative parameters were observed and compared between the two groups. The Visual Analogue Scale (VAS) score, Oswestry dysfunction index (ODI), Japanese Orthopaedic Association scores (JOA) and imaging parameters of the two groups were recorded and compared before the operation and during the follow-up.

**Results:**

A total of 53 patients with single segments and 21 patients with double segments were included in this study. During the follow-up, the VAS score, ODI index and JOA score of the two groups were significantly improved as compared with those before the operation(*P* < 0. 05). Ninety-two point five percent of single-segment patients and 90.5% of double segment patients returned to normal scoliosis within 12 months after the operation. The operation time, number of intraoperative fluoroscopy times and the amount of intraoperative blood loss in single-segment patients were better than those in double-segment group(*P* < 0. 05). At the last follow-up, the AVT, CBD and SVA in the double-segment group were 5.2 ± 2.3, 5.1 ± 1.0 and 12.2 ± 3.0 mm, respectively, which were higher than those in the single-segment group (1.9 ± 0.4, 1.1 ± 1.6 and 3.9 ± 2.1 mm) (*P* < 0. 05).

**Conclusion:**

PELD is an effective treatment for single-segment and double-segment lumbar disc herniation with Sciatic scoliosis. Double-segment patients can enjoy similar clinical efficacy to single-segment patients, avoiding complications caused by decompression, fusion, and internal fixation. Scoliosis was corrected spontaneously within 12 months after operation, and the sagittal curve was significantly improved in both groups. The improvement of coronal and sagittal balance in double -segment patients may take longer.

## Introduction

In addition to common low back pain and lower limb radiation pain, lumbar disc herniation can also be characterized by scoliosis and trunk list, namely sciatic scoliosis [1–3]. Studies have shown that the incidence of sciatic scoliosis in adult patients with lumbar disc herniation is approximately 1.4-32.0% [4–6], and sciatic scoliosis is a risk factor for poor prognosis of lumbar disc herniation [7]. With regard to its pathogenesis, scholars generally believe that sciatic scoliosis is a compensatory posture produced by the body to relieve nerve root stimulation, not structural scoliosis. After nerve root stimulation is eliminated, scoliosis can be corrected spontaneously [4, 8]. In recent years, PELD has been widely used and recognized in the treatment of lumbar disc herniation [9, 10]. Compared with traditional surgical methods, PELD has the advantages of high safety, less trauma, less bleeding, rapid postoperative recovery, little impact on nerve and spinal canal structure, and fewer postoperative complications [11, 12]. Previous studies have shown that PELD is an effective method for the treatment of lumbar disc herniation with sciatic scoliosis [13], but all of them are limited to single-segment lumbar disc herniation. There are few reports on the clinical effect of PELD in the treatment of double-segment patients. The double-segmental patients often receive decompression and fusion internal fixation because of many protruding segments and severe nerve compression. The clinical efficacy of PELD in the treatment of double-segment patients needs further study. If the clinical effect of double-segment patients is similar to that of single-segment patients, the complications such as adjacent spondylosis caused by decompression, fusion and internal fixation can be reduced. Therefore, the purpose of this study was to determine the difference in the incidence of single-segment and double-segment lumbar disc herniation with sciatic scoliosis, to evaluate the clinical efficacy of PELD in the treatment of adult single-segment and double-segment lumbar disc herniation with sciatic scoliosis, to compare the postoperative imaging features of single-segment and double-segment patients, and to further explore the improvement of postoperative function and the clinical outcome of scoliosis imaging parameters between the two groups.

## Methods

### General information

After obtaining the approval of the institutional Research Ethics Committee and the informed consent of the patients, the clinical data of 495 adult patients with lumbar disc herniation who received PELD from January 2019 to June 2022 were analyzed retrospectively. The inclusion criteria were as follows: (1) age of was 18–50 years old; (2) obvious lumbar symptoms accompanied by radiation pain or numbness in the legs, and there was no significant improvement after conservative treatment for more than one month. Physical examination found that the adam’s forward bend test was negative (nonstructural scoliosis); and (3) CT and MRI examinations suggested lumbar disc herniation, posterior dural sac or nerve root compression, and the segment of responsibility was consistent with symptoms and signs. (4) The X-ray findings of the standing orthopedic position showed that the medical records of Cobb angle ≥ 10 ° or Apical vertebral translation (AVT) ≥ 20 mm were complete and were followed up for more than 12 months. The exclusion criteria were as follows: (1) previous history of lumbar surgery; (2) lumbar spinal stenosis, lumbar spondylolisthesis or instability; (3) spinal infection or spinal tumor; (4) X-ray showed structural scoliosis, vertebral displacement or rotation; and (5) clinical manifestations such as low back pain or sciatica. Imaging examination showed no obvious abnormality. Finally, a total of 74 patients were included. According to the number of operative segments, the patients were divided into a single-segment group (*n* = 53) and a double-segment group (*n* = 21).

### Surgical strategies and methods

All patients were placed in the prone position and routinely sterilized with towels laid in the operation area. C-arm fluoroscopic localization was used before and during the operation. Then,3000 ml/ bag saline was used to wash continuously during the operation. The mode of operation depends on the surgical segment. Patients with lesion segment at or above L4-5 received Percutaneous Endoscopic Transforaminal Discectomy (PETD) under local anesthesia. Patients with lower iliac crest and segmental lesions located in L5-S1 received PETD under continuous epidural anesthesia. Patients with higher iliac crest and segmental lesions located in L5-S1 received percutaneous endoscopic interlaminar discectomy (PEID) under general anesthesia. The specific procedure was as follows:

- PETD: After the anesthesia took effect, the puncture needle was punctured to the shoulder of the superior articular process, and a soft tissue dilator was inserted step by step along the guide wire. Using a ring drill, the superior articular process was performed, the intervertebral foramen was enlarged, and the protruding nucleus pulposus was removed by an intervertebral foramen endoscope.
- PEID: After anesthesia, the puncture needle was punctured into the interlaminar space of L5-S1, and the soft tissue dilator and working cannula were placed step by step along the guide wire. Use a gun-shaped rongeur to enlarge the interlaminar space appropriately. After exposing the dural sac and nerve root, the protruding nucleus pulposus tissue was removed and the nerve root was fully decompressed on the shoulder and axilla.

The effect of nerve root decompression was satisfactory in all patients, and the radiofrequency knife head was used for hemostasis and annulus closure. Both groups were treated with dehydration, detumescence and infection prevention after the operation. The patients were instructed to get out of bed gradually under the protection of a lumbar brace on the second day after the operation. The lumbar brace was worn for 1 month, and strenuous activity was avoided within 3 months.

### Observation indicators

The perioperative data such as operation time, intraoperative fluoroscopy times, intraoperative blood loss, postoperative hospital stay and complications of the two groups were analyzed retrospectively. Pfirrmann grading was used to evaluate the degree of intervertebral disc degeneration. At 1/3/12 months and the last follow-up, Visual Analogue Scale (VAS) score was used to evaluate the degree of low back and leg pain, Oswestry Dysfunction Index (ODI) and Japanese Orthopaedic Association Scores (JOA) were used to evaluate the quality of life, and the improved Macnab standard was used to evaluate the treatment effect. The coronal and sagittal parameters of the whole spine were measured during follow-up. Standardize the radiological examination program: patients stand in a comfortable position, stretch their hips and knees, relax their arms, and place their hands on shoulder-level support [14]. Double experienced physicians used Surgimap software (version 2.3.2.1) to measure imaging parameters independently. The average value of three times of measurement is taken as the measurement result, and the measurement accuracy is 0.1 °or 0.1 mm. The average value of the measurement results of 2 doctors was taken as the final value for statistical analysis. The specific parameters are as follows:

- *Cobb angle*: angle of intersection between the vertical line of the upper edge of the cephalic end of the coronal vertebra and the vertical line of the lower edge of the caudal end;
- *Apical vertebral translation*(AVT): the horizontal distance from the midpoint of the parietal vertebra to the transsacral midline in coronal scoliosis;
- *Coronal balance distance*(CBD): the horizontal distance from the vertical line of C7 to the median sacral line in the coronal position;
- *Sagittal vertical axis*(SVA): the horizontal distance between the sagittal plumb line passing through the midpoint of C7 vertebra and the posterior superior border of S1.
- *Thoracic kyphosis*(TK): the angle between the superior endplate of T5 and the inferior endplate of T12 in sagittal position;
- *Lumbar lordosis*(LL): the angle between the superior endplate of T12 and S1 in sagittal position;
- *Pelvic Incidence*(PI): sagittal angle between the midpoint of the superior S1 endplate and the central line of the femoral head and the vertical line of the superior S1 endplate passing through the midpoint of the superior S1 endplate (if the bilateral femoral heads do not overlap, take the midpoint of the line connecting the center of the two femoral heads).

Changes in AVT values and resolution rate (RR) were used to assess the evolution of scoliosis. The previous literature definition of scoliosis return to normal [15]: AVT ≤ 10 mm. RR is defined as the ratio of scoliosis to normal.

### Statistical analysis

The Shapiro-Wilk test was used for the normality test, and measurement data in accordance with a normal distribution were expressed as the mean ± standard deviation. A paired sample t-test was used for comparison of patients in the same group before and after operation, and an independent sample t-test was used for comparison between the two groups. The count data are expressed as the rate (%), and the difference was statistically significant by the chi-square test. The difference was statistically significant (*P* < 0.05).

## Results

### General information

A total of 74 patients (male/female: 33/41) were enrolled in this study. The average age was 35.2 ± 11.4 years, the average Body Mass Index(BMI)was 24.9 ± 4.0 kg/m^2^, the average duration of symptoms was 6.0 ± 2.9 months, and the average follow-up time was 18.2 ± 6.9 months. According to the number of operative segments, the patients were divided into a single-segment group and double-segment group. 53 patients were included in the single-segment group and 21 patients were included in the double-segment group. There was no significant difference in sex, age, BMI index, symptom duration or follow-up time between the two groups (*P*>0.05). In the single-segment group, the number of patients with protruding segments located in L4-5, L5-S1 and other segments was 25, 20 and 8 respectively, the number of central and paracentric patients was 10 and 43 respectively, and the number of patients with Pfirrmann grades II, III and IV was 11, 22 and 20 respectively. In the double-segment group, the numbers of patients with protruding segments located in L4-5, L5-S1 and other segments were 18 and 3, respectively, the numbers of central and paracentric patients were 4 and 17 respectively, and the number of patients with Pfirrmann grades II, III and IV were 4, 7 and 10, respectively. The proportion of patients with Pfirrmann grade IV in the double-segment group was higher than that in the single-segment group (*P* < 0.05) (Table 1).

**Table 1:** Demographic and clinical characteristics for all the cases

	Single-segment group	Double-segment group
Number of cases (n)	53	21
Sex (male/female)	25/28	8/13
Age (years)	35.9 ± 11.8	34.4 ± 12.1
BMI (kg/m^2^)	24.7 ± 3.5	25.8 ± 4.2
Mean Duration of Symptoms (months)	6.2 ± 2.7	5.9 ± 3.1
Follow-up duration (months)	18.1 ± 6.1	18.3 ± 7.5
Herniated Level	L4-5/L5-S1/ Otherwise :25/20/8	L4-5,L5-S1/ Otherwise: 18/3
Disc location(Central/ Paramedian)	10/43	4/17
Pfirrmann grades(II/III/IV)	11/22/20	4/7/10

### Perioperative parameters

The operation time was 65.4 ± 23.5 min in the single-segment group and 110.6 ± 19.4 min in the double-segment group. Intraoperative fluoroscopy was performed 5.2 ± 2.1 times in the single-segment group and 8.9 ± 3.4 times in the double-segment group. The intraoperative blood loss was 5.1 ± 3.8 ml in the single-segment group and 9.4 ± 4.2 ml in the double-segment group. The postoperative hospital stay was 2.3 ± 1.1 days in the single-segment group and 2.6 ± 1.2 days in the double-segment group. The operation time, intraoperative fluoroscopy times and intraoperative blood loss in the single-segment group were lower than those in the double-segment group (*P* < 0.05). There was no significant difference in postoperative hospital stay between the two groups (*P* > 0.05) (Table 2).

**Table 2:** Perioperative parameters

Parameters	Single-segment group	Double-segment group
Operation time(min)	65.4 + 23.5	110.6 + 19.4^a^
Intraoperative fluoroscopy times(time)	5.2 ± 2.1	8.9 ± 3.4^a^
Amount of intraoperative blood loss(ml)	5.1 ± 3.8	9.4 ± 4.2^a^
Postoperative hospital stay(day)	2.3 ± 1.1	2.6 ± 1.2

^a^Compared with single-segment group, the difference was significant (*P* < 0.05)

### Clinical effect

Both groups completed the operation successfully and were followed up for at least 12 months. No serious complications such as cerebrospinal fluid leakage, infection or intraspinal hematoma occurred in 74 cases. Within one week after the operation, hip pain occurred in 2 patients in the single-segment group, and the symptoms disappeared after hot compress and local closure treatment. One month after the operation, there was one case of recurrent lumbar disc herniation at the same segment in the single-segment group and double-segment group, and the symptoms were relieved by transforaminal interbody fusion. Before operation, 1 month, 3 months, 12 months after operation, and at the last follow-up, the back VAS scores of single-segment group were 7.2 ± 1.3,2.5 ± 1.2,2.3 ± 1.8,1.9 ± 1.1,1.7 ± 1.0, respectively, while those of double-segment group were 7.9 ± 1.6,3.0 ± 1.2,1.6 ± 1.2,1.4 ± 1.1,1.3 ± 1.2, respectively. The leg VAS scores of the single-segment group were 7.0 ± 1.2,3.6 ± 1.3,2.2 ± 1.4,1.8 ± 1.2,1.3 ± 1.1, respectively, while those of double-segment group were 6.8 ± 1.5, 2.9 ± 1.5, 1.9 ± 1.1, 1.7 ± 1.0, 1.2 ± 0.8, respectively. The ODI index of the single-segment group was 44.9 ± 3.8, 12.2 ± 2.8, 11.0 ± 4.1, 7.9 ± 3.4, and 6.8 ± 3.5, respectively. The ODI index of the double-segment group was 47.1 ± 4.2%, 12.6 ± 2.5%, 11.5 ± 3.3%, 8.9 ± 3.4%, and 7.9 ± 3.0%, respectively. The JOA scores of the single-segment group were 12.5 ± 3.7, 21.9 ± 3.2, 23.0 ± 3.5, 25.2 ± 4.1, and 26.2 ± 3.8, respectively. The JOA scores of the double -segment group were 12.6 ± 3.4, 22.6 ± 3.4, 23.1 ± 4.6, 25.6 ± 3.7, and 26.3 ± 3.5 respectively. During the follow-up, the back VAS score, leg VAS score, ODI index and JOA score were significantly improved in the two groups (*P* < 0.05). There was no significant difference between the two groups before the operation or at the same follow-up time (*P*>0.05). The excellent and good rates of modified Macnab at 1 month and 3 months after operation in the single-segment group were 96.2% and 98.1% respectively, and the rest of the follow-up time was 100%. One month after the operation, the excellent and good rate of modified Macnab in the double-segment group was 95.2%, and the rest of the follow-up time was 100%. There was no significant difference between the two groups (*P*>0.05) (Tables 3 and 4).

**Table 3:** Clinical outcomes of patients

Outcome	Single-segment group					Double-segment group				
	Preoperative	Postoperative				Preoperative	Postoperative			
		1 month	3 months	12 months	Final follow-up		1 month	3 months	12 months	Final follow-up
VAS Back Pain	7.2 ± 1.3	2.5 ± 1.2*	2.3 ± 1.8*	1.9 ± 1.1*	1.7 ± 1.0*	7.9 ± 1.6	3.0 ± 1.2*	1.6 ± 1.2*	1.4 ± 1.1*	1.3 ± 1.2*
VAS Leg Pain	7.0 ± 1.2	3.6 ± 1.3*	2.2 ± 1.4*	1.8 ± 1.2*	1.3 ± 1.1*	6.8 ± 1.5	2.9 ± 1.5*	1.9 ± 1.1*	1.7 ± 1.0*	1.2 ± 0.8*
ODI(%)	44.9 ± 3.8	12.2 ± 2.8*	11.0 ± 4.1*	7.9 ± 3.4*	6.8 ± 3.5*	47.1 ± 4.2	12.6 ± 2.5*	11.5 ± 3.3*	8.9 ± 3.4*	7.9 ± 3.0*
JOA	12.5 ± 3.7	21.9 ± 3.2*	23.0 ± 3.5*	25.2 ± 4.1*	26.2 ± 3.8*	12.6 ± 3.4	22.6 ± 3.4*	23.1 ± 4.6*	25.6 ± 3.7*	26.3 ± 3.5*

*Compared with preoperative, the difference was significant (*P* < 0.05)

**Table 4:** Modified Macnab criteria for patients’ satisfaction

	Single-segment group				Double-segment group			
	1 month	3 months	12 months	Final follow-up	1 month	3 months	12 months	Final follow-up
Excellent	28	35	41	48	8	11	16	19
Good	23	17	12	5	12	10	5	2
Fair	2	1	0	0	1	0	0	0
Poor	0	0	0	0	0	0	0	0

### Imaging parameters

**Fig. 1:**
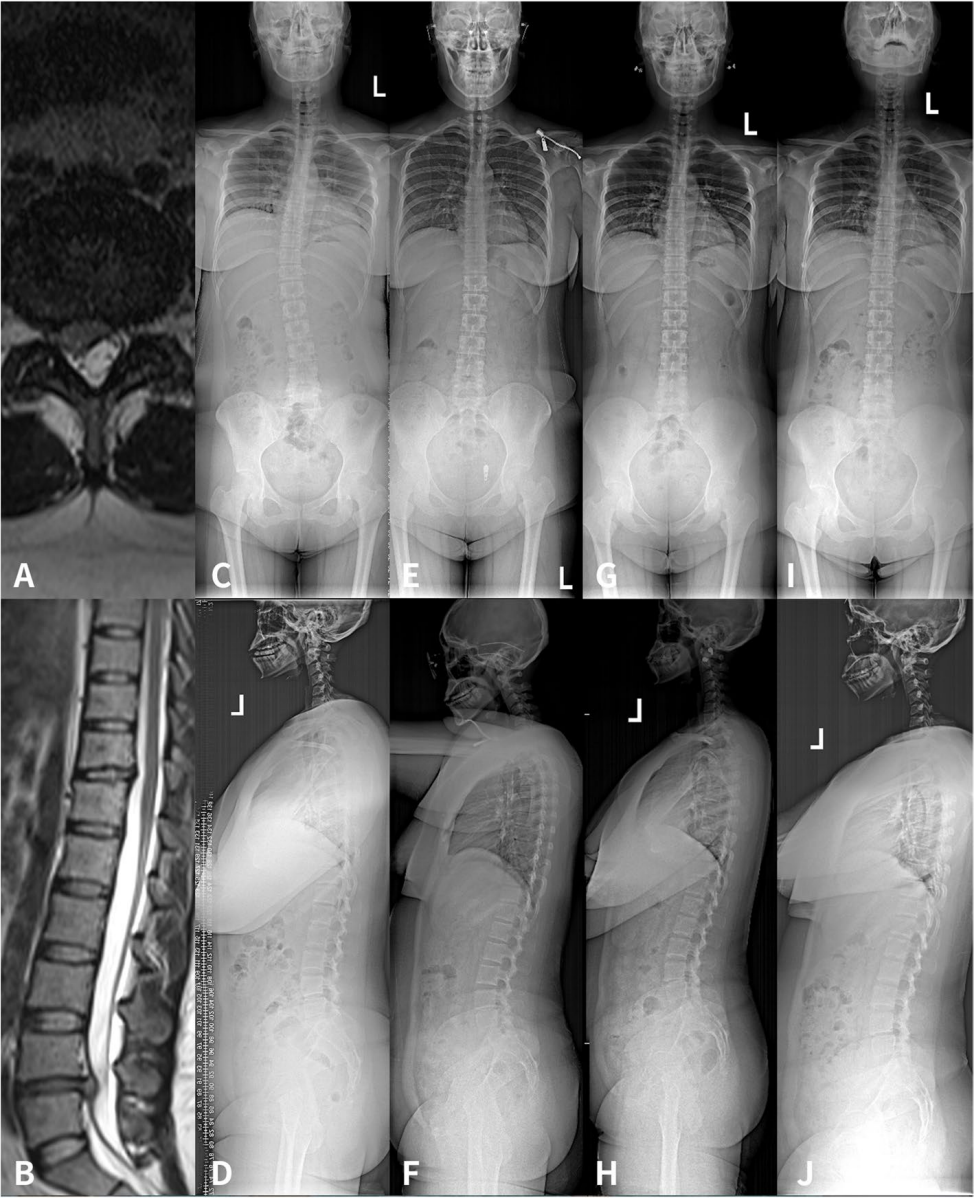
A 36-year-old female was admitted with “recurrent low back pain and right lower limb pain for more than half a year”. She was diagnosed as lumbar disc herniation at L4/5 and was treated with PELD in our hospital. A-D were preoperative lumbar magnetic resonance and anterior and lateral X-ray of the whole spine, Cobb angle: 17.5 °; AVT:22.6 mm, CBD:5.5 mm, SVA:11.4 mm, TK:11.8 °, LL:44.6 °, PI:39.7 °; E-F: postoperative anterior and lateral X-ray of the whole spine, Cobb angle: 5.7 °, AVT:16.7 mm, CBD:5.3 mm, SVA:37.5 mm, TK:13.2 °, LL:48.1 °. G-H were positive and lateral X-ray of the whole spine at 1 month follow-up, Cobb angle was 2.3°, AVT:7.8 mm, CBD:4.9 mm, SVA:35.0 mm, TK:14.1 °, LL:50.7 °, I-J Cobb angle 2.1°, AVT:5.7 mm, CBD:4.1 mm, SVA:29.1 mm, TK:15.9 °, LL:55.2°

**Fig. 2:**
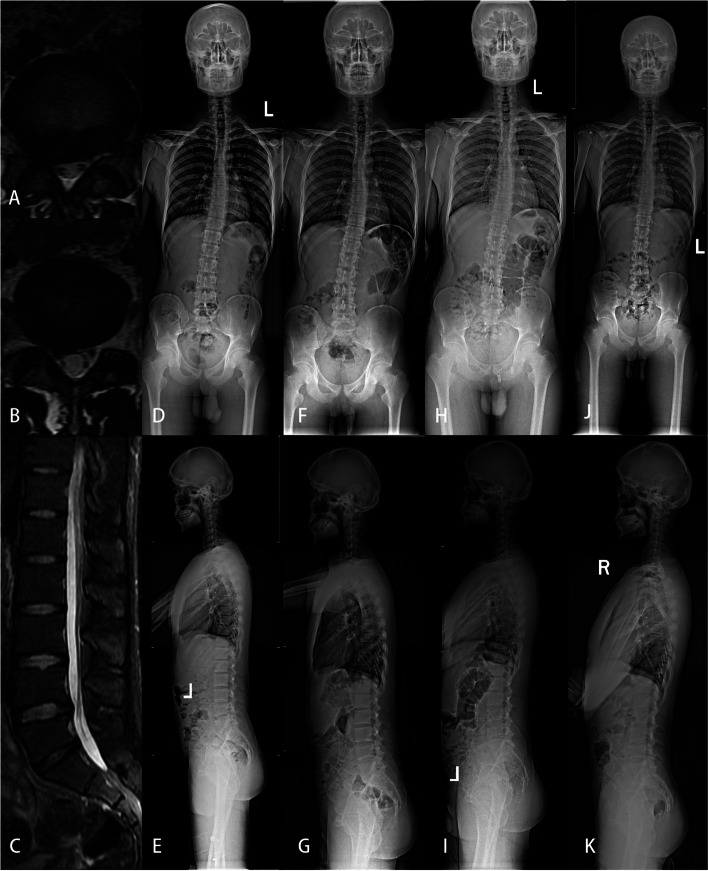
A 24-year-old male was admitted with “recurrent low back pain and right lower limb pain for more than one year”. He was diagnosed as lumbar disc herniation at L4/5 and L5/S1, and was treated with PELD in our hospital. A-E were preoperative lumbar magnetic resonance and whole spinal anteroposterior and lateral X-ray, A was L4/5 cross section, B was L5/S1 cross section, Cobb angle was 15.2 °; AVT:46.4 mm, CBD:54.2 mm, SVA:85.6 mm, TK:16.6 °, LL:41.4 °, PI:48.6 °; F-G were postoperative whole spinal positive and lateral X-ray, Cobb angle was 9.3°; AVT:45.4 mm, CBD:52.3 mm. SVA:84.1 mm, TK:17.7 °, LL:49.1 °; H-I: Cobb angle: 5.8 mm, AVT:39.7 mm, CBD: 40.5 mm, SV-A: 34.3 mm; TK: 17.8 mm, LL:49.4 °; J-K: Cobb angle: 3.9°, AVT:18.9 mm, CBD:28.7 mm, SVA:-2.2 mm, TK:20.1 °, LL:50.5 °

The coronal and sagittal imaging parameters of the two groups are shown in Table 5. The average PI of the single-segment group was 48.5°, and that of the double-segment group was 37.2°. PI was considered to be a fixed parameter. Before the operation, at 1 month, 3 months, and 12 months and at the last follow-up, the Cobb angle of the single-segment group was 15.3 ± 2.8°, 8.5 ± 2.9°, 5.7 ± 2.5°, 3.1 ± 1.0°, and 2.4 ± 0.6°, and the Cobb angle of the double-segment group was 15.7 ± 4.6°, 7.9 ± 3.3°, 6.0 ± 2.5°, 2.7 ± 1.5°, and 2.2 ± 1.0°, respectively. The AVT of single-segment group was 25.6 ± 15.1 mm, 8.9 ± 2.9 mm, 5.8 ± 3.4 mm, 5.4 ± 2.7 mm, 1.9 ± 0.4 mm, the AVT of double-segment group was 28.9 ± 13.4 mm, 9.7 ± 3.2 mm, 6.1 ± 2.5 mm, 5.5 ± 1.9 mm, 5.2 ± 2.3 mm. The CBD of the single-segment group was 19.0 ± 3.1 mm, 8.1 ± 3.3 mm, 5.3 ± 3.2 mm, 1.4 ± 2.1 mm, and 1.1 ± 1.6 mm. The CBD of the double-segment group was 20.3 ± 2.7 mm, 8.5 ± 3.0 mm, 5.4 ± 3.8 mm, 5.2 ± 1.8 mm, and 5.1 ± 1.0 mm. The SVA of single-segment group was 50.1 ± 21.8 mm, 22.8 ± 12.4 mm, 17.1 ± 9.4 mm, 6.0 ± 3.3 mm, 3.9 ± 2.1 mm, and the SVA of double-segment group was 49.7 + 18.2 mm, 21.9 ± 13.0 mm, 16.4 ± 10.3 mm, 13.6 ± 3.4 mm, 12.2 ± 3.0 mm, respectively. The TK of the single-segment group was 12.8 ± 8.2°, 13.8 ± 7.6°, 16.1 ± 7.9°, 25.3 ± 9.1°, and 30.1 ± 13.4°, and the TK of the double-segment group was 11.9 ± 7.1°, 12.3 ± 8.4°, 15.5 ± 7.2°, 26.0 ± 8.6°, and 31.2 ± 8.0°. The LLs of the single-segment group were 35.9 ± 8.6°, 37.2 ± 7.9°, 41.3 ± 10.1°, 49.6 ± 8.7°, and 51.9 ± 10.5°, respectively. The LLs of the double-segment group were 36.8 ± 8.4°, 38.4 ± 8.0°, 40.5 ± 9.6°, 50.6 ± 9.4°, and 53.2 ± 9.4°. Before the operation, the PI of the single-segment group was higher than that of the double-segment group (*P* < 0.05), but there was no significant difference in other imaging parameters (*P*>0.05). There was no significant difference in TK, LL at 1 month and TK at 3 months after operation in the single-segment group (*P* > 0.05), and there was no significant difference in TK and LL at 1 month and 3 months after operation in the double-segment group (*P* > 0.05). During the rest of the follow-up, the Cobb angle, AVT, CBD, SVA, TK and LL were significantly improved in both groups (*P* < 0.05). CBD, SVA at 12 months after operation and AVT, CBD and SVA at the last follow-up in the double-segment group were higher than those in the single-segment group (*P* < 0.05). At 1 month, 3 months, 12 months and the last follow-up, the resolution rate and RR of the single-segment group were 50.9%, 75.5%, 92.5% and 94.3%, respectively, and those of the double-segment group were 47.6%, 71.4%, 90.5% and 95.2%, respectively. Typical cases are shown in Figs. 1 and 2.

**Table 5:** Radiological results of patients

Measures	Single-segment group					Double-segment group				
	Preoperative	Postoperative				Preoperative	Postoperative			
		1 month	3 months	12 months	Final follow-up		1 month	3 months	12 months	Final follow-up
PI(°)	48.5	-	-	-	-	37.2^#^	-	-	-	-
Cobb angle (°)	15.3 ± 2.8	8.5 ± 2.9*	5.7 ± 2.5*	3.1 ± 1.0*	2.4 ± 0.6*	15.7 ± 4.6	7.9 ± 3.3*	6.0 ± 2.5*	2.7 ± 1.5*	2.2 ± 1.0*
AVT(mm)	25.6 ± 15.1	8.9 ± 2.9*	5.8 ± 3.4*	5.4 ± 2.7*	1.9 ± 0.4*	28.9 ± 13.4	9.7 ± 3.2*	6.1 ± 2.5*	5.5 ± 1.9*	5.2 ± 2.3*^#^
CBD(mm)	19.0 ± 3.1	8.1 ± 3.3*	5.3 ± 3.2*	1.4 ± 2.1*	1.1 ± 1.6*	20.3 ± 2.7	8.5 ± 3.0*	5.4 ± 3.8*	5.2 ± 1.8*^#^	5.1 ± 1.0*^#^
SVA(mm)	50.1 ± 21.8	22.8 ± 12.4*	17.1 ± 9.4*	6.0 ± 3.3*	3.9 ± 2.1*	49.7 + 18.2	21.9 ± 13.0*	16.4 ± 10.3*	13.6 ± 3.4*^#^	12.2 ± 3.0*^#^
TK(°)	12.8 ± 8.2	13.8 ± 7.6	16.1 ± 7.9	25.3 ± 9.1*	30.1 ± 13.4*	11.9 ± 7.1	12.3 ± 8.4	15.5 ± 7.2	26.0 ± 8.6*	31.2 ± 8.0*
LL(°)	35.9 ± 8.6	37.2 ± 7.9	41.3 ± 10.1*	49.6 ± 8.7*	51.9 ± 10.5*	36.8 ± 8.4	38.4 ± 8.0	40.5 ± 9.6	50.6 ± 9.4*	53.2 ± 9.4*
RR(%)	-	50.9	75.5	92.5	94.3	-	47.6	71.4	90.5	95.2

*Compared with preoperative, the difference was significant (*P* < 0.05)

^#^Compared with single-segment group at the same time, the difference was significant (*P* < 0.05)

## Discussion

Patients with lumbar disc herniation may have sciatic scoliosis, which is called nonstructural scoliosis secondary to nerve root irritation. Scholars generally believe that this is the compensatory behavior produced by the body to alleviate nerve root stimulation [3, 16], and some scholars have pointed out that the hyperactivity of paraspinal muscles is related to the occurrence of sciatic scoliosis [17–19].

There are different reports on the incidence of sciatic scoliosis in patients with lumbar disc herniation. Kim et al. [5] reported that the incidence of sciatic scoliosis in 164 adult patients with lumbar disc herniation was approximately 18%. Ozgen et al. [20] and other reports pointed out that the incidence of sciatic scoliosis is higher in adolescent patients with lumbar disc herniation, approximately 47%. Zhang et al. [15] reported that among 1087 patients with lumbar disc herniation, the incidence of sciatic scoliosis in adolescents and adults was approximately 23.8% and 12.2%, respectively. There is no report on the incidence of sciatic scoliosis in patients with single-segment and double-segment lumbar disc herniation. In this study, a retrospective analysis of 495 patients with lumbar disc herniation was conducted, and a total of 74 cases were found to have sciatic scoliosis, with an overall incidence of 14.9%. Among them, 53 and 21 patients had single-segment and double-segment lumbar disc herniation, respectively, and the incidence of single-segment and double-segment lumbar disc herniation with sciatic scoliosis was 10.7% and 4.2%, respectively. The incidence in double-segment patients is much lower than that in single-segment patients.

Kim et al. [5] reported that L4-5 intervertebral disc herniation is a risk factor for sciatic scoliosis. In this study, sciatic scoliosis was more common in patients with L4-5 lumbar disc herniation, which is consistent with previous reports [5, 8] and may be related to the anatomical characteristics of L4-5. The bilateral iliolumbar ligament starts from the transverse process of L4 and L5, ends at the sacroiliac joint and iliac crest, restricts the movement of the vertebral body and maintains its stability. However, L4-5 is not limited to the pelvic cavity and is more active than L5, which leads to greater shear force and longitudinal pressure in L4-5, and is more prone to degeneration. Therefore, L4-5 intervertebral disc herniation is more likely to be secondary to sciatic scoliosis.

For patients with ineffective conservative treatment, surgical treatment is the first choice [21–24]. Compared with traditional open surgery, PELD has the advantages of avoiding excessive exposure of the nerve root, less bone resection, less injury to the facet joint and muscle ligament structure, a low postoperative infection rate, and reducing the incidence of postoperative complications such as adjacent spondylosis caused by traditional open surgery [11, 25–29]. In addition, PELD can ablate the new blood vessels and granulation tissue after the rupture of the annulus fibrosus to reduce the inflammatory response [30].

Previous studies have reported that the clinical effect of PELD is similar to that of open surgery [9]. However, previous studies were limited to single-segment patients, and there was no report on the curative effect of double-segment patients. Double-segment lumbar disc herniation with sciatic scoliosis is often considered as a contraindication of PELD because of its many protruding segments, severe nerve compression and difficult operation. Double-segment patients often receive traditional open decompression and fusion internal fixation, which requires full dissection of paraspinal muscles, resection of some articular processes and bone grafting and fusion of the responsible segment, which is invasive and can lead to many complications such as bleeding during operation, intractable low back pain after operation, back muscle injury and accelerated degeneration of adjacent segments [31]. The clinical effect of PELD on double -segment patients needs further study.

In this study, during the follow-up of the two groups, the back VAS score, leg VAS score, JOA score and ODI index were significantly improved compared with those before the operation. Sciatic scoliosis patients have the characteristics of a relatively straight sagittal position, reduced thoracic kyphosis and lumbar kyphosis, and some patients with sagittal imbalance [32]. According to previous studies [6], sagittal imbalance is defined as SVA ≥ 40 mm. In this study, the preoperative TK and LL of single-segment patients were 12.8 ± 8.2° and 35.9 ± 8.6°, respectively, and the preoperative TK and LL of double-segment patients were 11.9 ± 7.1° and 36.8 ± 8.4°, respectively. The preoperative SVA of the two groups was 50.1 ± 21.8 mm and 49.7 ± 18.2 mm, respectively. After PELD treatment, at the last follow-up, the TK and LL of single-segment patients improved to 30.1 ± 13.4° and 51.9 ± 10.5°, respectively, and the TK and LL of double-segment patients improved to 31.2 ± 8.0° and 53.2 ± 9.4°, respectively. The SVA of the two groups improved to 3.9 ± 2.1 mm and 12.2 ± 3.0 mm, respectively. The sagittal imaging parameters were significantly improved compared with those before the operation, and all patients reached the standard of sagittal balance. This shows that after PELD treatment, nerve compression is relieved, the sagittal curve can be changed, and thoracic kyphosis and lumbar kyphosis can be increased. However, the patients in the double-segment group had a longer operation time, more intraoperative fluoroscopy and more intraoperative bleeding. During the follow-up, there was no significant difference in back VAS score, leg VAS score, JOA score or ODI index between the two groups at the same follow-up time. This shows that PELD can achieve satisfactory short-term effects and long-term effects for both single-segment and double-segment lumbar disc herniation with sciatic scoliosis. PELD for double-segment patients can reduce the related complications caused by traditional decompression, fusion and internal fixation.

This study found that at the last follow-up, both groups reached the coronal and sagittal balance criteria, but the AVT, CBD and SVA in the double-segment group were higher than those in the single-segment group, indicating that the long-term recovery of coronal and sagittal balance in the double-segment group was worse than that in the single-segment group. The author believes that this may be related to the lower PI of the double-segment group in this study. PI angle is a key anatomical parameter for the stability of spine-pelvic sagittal plane, which is related to sacral inclination and spinal curvature. In theory, patients with small PI angle are less able to compensate for the imbalance [33]. In the double-segment group, the PI value was low, the range of pelvic rotation around the femoral head was also lower, the pelvic parameters were smaller, the lumbar kyphosis was relatively flat, the center of gravity moved forward, and the compensation ability of sagittal pelvic retroversion was limited [34–36]. The self-compensating ability of the spine and pelvis to the curvature changes caused by lumbar degenerative diseases was weak, and the recovery of sagittal balance was poor after nerve compression was relieved. In addition, in this study, the proportion of patients with preoperative Pfirrmann grade IV in double-segment patients was higher than that in single-segment patients, which indicated that in double-segment patients, there were more serious degeneration of intervertebral disc, more imbalance of extracellular matrix catabolism, less number of nucleus pulposus cells, more apoptosis, lower cell density, active proliferation and functional protein synthesis, and lower proteoglycan, type II collagen and water content [37]. Type I and III collagen fibers are higher, resulting in more loss of intervertebral disc height, lower elasticity and tension of intervertebral disc, smaller nerve root volume, stronger chronic inflammatory reaction and more serious intervertebral instability [38, 39]. The intervertebral disc is not enough to support the large-scale activity of the patient, which is not conducive to the recovery and reconstruction of the coronal and sagittal plane after operation.

In addition to pain relief, patients are often concerned about whether the scoliosis posture can be corrected. Previous studies have shown that sciatic scoliosis is reversible. After nerve compression is released, the scoliosis posture can be corrected by itself. Zhang et al. [15] reported that six months after surgery, the resolution rates (RRs) of scoliosis in adolescents and adults were 85.71% and 92.68%, respectively. Kim et al. [5] and other studies have shown that 6 months after surgery, the resolution rate of scoliosis is more than 50%. Tu et al. [13] performed a retrospective analysis of 42 patients with sciatic scoliosis and found that the average Cobb angle improved from 18.4° to 8.7° three months after the operation. In this study, at the last follow-up, the average Cobb angle of single-segment group and double-segment group were 2.4 ± 0.6 °and 2.2 ± 1.0 °, AVT were 1.9 mm ± 0.4 mm and 5.2 ± 2.3 mm, CBD were 1.1 mm ± 1.6 and 5.1 mm ± 1.0 mm, respectively, which were significantly better than those before operation. At the last follow-up, scoliosis subsided in 94.3% of single-segment patients and 95.2% of double-segment patients. Early operation is beneficial to the correction of scoliosis posture and can avoid the development of sciatic scoliosis to structural scoliosis.

There are some limitations in this study. In the process of imaging parameter measurement, there may be some measurement bias due to the existence of osteophytes or the quality of X-ray films. The curve recovery of sciatic scoliosis is a dynamic process, and the 12-month follow-up cannot fully reflect the clinical outcome of patients after PELD. The effects of PI and the degree of intervertebral disc degeneration on the recovery of coronal and sagittal curves need to be further studied.

## Conclusions

In summary, the incidence of single-segment lumbar disc herniation with sciatic nerve scoliosis is higher than that of double-segment patients, and L4-5 is the common segment. The efficacy of PELD is similar in single- and double -segment patients. After nerve compression is relieved, the scoliosis posture can be self-corrected, and the sagittal curve can be changed. Double-segment patients may avoid internal fixation with decompression fusion .The shape of the pelvis and the degree of intervertebral disc degeneration have a certain influence on the recovery of coronal and sagittal balance of patients, and the recovery time of patients with low PI and severe intervertebral disc degeneration may be longer.

## Data Availability

The datasets generated and analysed during the current study are not publicly available due restrictions on ethical approvals involving patient data and anonymity but are available from the corresponding author on reasonable request.

## Abbreviations

PELD: Percutaneous endoscopic lumbar discectomy
VAS: Visual Analogue Scale
ODI: Oswestry dysfunction index
JOA: Japanese Orthopaedic Association Scores
PETD: Percutaneous Endoscopic Transforaminal Discectomy
PEID: Percutaneous Endoscopic Interlaminar Discectomy
AVT: Apical vertebral translation
CBD: Coronal Balance Distance
SVA: Sagittal Vertical Axis
TK: Thoracic Kyphosis
LL: Lumbar Lordosis
PI: Pelvic Incidence
RR: Resolution Rate
BMI: Body Mass Index
